# Effects of stocking density on the homeostasis of uric acid and related liver and kidney functions in ducks

**DOI:** 10.5713/ab.23.0364

**Published:** 2024-01-20

**Authors:** Peiyi Lin, Sui Liufu, Jinhui Wang, Zhanpeng Hou, Yu Liang, Haiyue Wang, Bingxin Li, Nan Cao, Wenjun Liu, Yunmao Huang, Yunbo Tian, Danning Xu, Xiujin Li, Xinliang Fu

**Affiliations:** 1 College of Animal Science and Technology, Zhongkai University of Agriculture and Engineering, Guangzhou 510225, China; 2 Waterfowl Healthy Breeding Engineering Research Center of Guangdong, Guangzhou, 510225, China

**Keywords:** Hyperuricemia, Inflammatory Lesions, Stocking Density, Uric Acid

## Abstract

**Objective:**

Stocking density (SD) is an important issue in the poultry industry, which is related to the production performance, intestinal health and immune status. In the present study, the effects of SD on the metabolism and homeostasis of uric acid as well as the related functions of the liver and kidney in ducks were examined.

**Methods:**

A total of 360 healthy 56-day-old Shan-ma ducks were randomly divided into the low stocking density (n = 60, density = 5 birds/m^2^), medium stocking density (n = 120, density = 10 birds/m^2^) and high stocking density groups (HSD; n = 180, density = 15 birds/m^2^). Samples were collected in the 3rd, 6th, and 9th weeks of the experiment for analysis.

**Results:**

The serum levels of uric acid, lipopolysaccharide and inflammatory cytokines (interleukin-1β [IL-1β], IL-8, and tumor necrosis factor-α [TNF-α]) were increased significantly in the HSD group. Serious histopathological lesions could be seen in both the livers and kidneys in the HSD group in the 9th week. The mRNA expression levels of inflammatory cytokines (IL-8 and TNF-α) and related pathway components (toll-like receptor 4, myeloid differentiation primary response gene 88, and nuclear factor-κB) were increased significantly in both the livers and kidneys in the HSD group. The mRNA expression levels of enzymes (adenosine deaminase, xanthine oxidase, phosphoribosyl pyrophosphate amidotransferase, and phosphoribosyl pyrophosphate synthetase 1) related to the synthesis of uric acid increased significantly in the livers in the HSD group. However, the mRNA expression level of solute carrier family 2 member 9, which plays an important role in the excretion of uric acid by the kidney, was decreased significantly in the kidneys in the HSD group.

**Conclusion:**

These results indicated that a higher SD could cause tissue inflammatory lesions in the liver and kidney and subsequently affect the metabolism and homeostasis of uric acid, and is helpful for guiding decisions related to the breeding and production of ducks.

## INTRODUCTION

With the development of the modern intensive poultry industry, stocking density (SD) has become one of the most important environmental and management factors; SD refers to the density of poultry in a specific breeding space [1]. Increasing SD properly could enhance productivity, save production costs and increase the output of production. However, if the SD exceeds the proper range, it has negative effects on the health, welfare and performance of poultry, causing poor growth performance and intestinal microbial diversity disruption. Studies on the effects of SD on poultry health and production have been carried out in recent years. It has been documented that high SD decreased feed intake, body weight, weight gain, and feed conversion in chickens [1,2]. Several studies have also reported that increasing SD adversely affected the growth performance and meat yield of broilers [3,4]. In addition, stress from high SD negatively affected gut morphology and microbial structure and composition [5,6]. Furthermore, high SD increased physiological and oxidative stress levels and decreased immunity and absorptive capacity by impairing villus structures of the small intestine in broiler chickens [7–9]. The effects of SD in ducks, which cause growth depression, a decrease in breast and leech meat yield, inflammation and chronic liver diseases, have also been reported [10,11]. However, the relationship between SD and the metabolism of uric acid (UA) in ducks has not been clarified. Moreover, there is no consensus regarding the appropriate SD for large-scale poultry [12].

Avian gout is a common metabolic disease characterized by hyperuricemia (HUA) and urate deposits in the joints, and it is caused by the overproduction of UA by hepatic metabolism or renal underexcretion [13]. The processes of UA synthesis in the liver and excretion in the kidney are the primary factors that affect the level of UA in serum, and the increase in UA could lead to HUA and further induce avian gout. UA is the final product of purine metabolism in poultry, and a series of enzymes are responsible for its synthesis, including adenosine deaminase (ADA), xanthine oxidase (XOD), phosphoribosyl pyrophosphate amidotransferase (PRPPAT), and phosphoribosyl pyrophosphate synthetase 1 (PRPS1) in the liver [14,15]. The increase in the activities and mRNA expression levels of the enzymes above could lead to the overproduction of UA, which is the main cause of HUA and an important factor that causes gout [14,16,17]. In addition, renal excretion of UA requires coordination of various UA transporters, which is key to renal regulation of the homeostasis of UA [18]. ATP-binding cassette subfamily G member 2 (ABCG2) and solute carrier family 2 member 9 (SLC2A9) in the kidney have been reported to play important roles in the regulation of UA [16,19], and the SLC2A9 pathway might be the key pathway of urate reabsorption and excretion from the proximal tubule in the kidney [15,20]. Approximately two-thirds of UA is excreted by the kidney, and the underexcretion of UA by the kidney is the most important cause of HUA and gout [21]. Currently, avian gout occurs frequently among not only broilers and goslings but also ducks in the poultry breeding industry, which has attracted attention in recent years [17,22,23]. The level of UA is the core indicator leading to gout; therefore, this study aimed to determine whether SD affects the metabolism and homeostasis of UA and the related functions of the liver and kidney in ducks, which is helpful for the prevention of avian gout, as well as the breeding and production of ducks.

## MATERIALS AND METHODS

### Animal care and ethics statement

All experimental work was conducted in accordance with the recommendations in the Guide for the Institutional Animal Care and Use committee of Zhongkai University of Agriculture and Engineering (NO.2020071109). The protocol was approved by the Committee on the Ethics and Welfare of Animal Experiments of Zhongkai University of Agriculture and Engineering.

### Animals, diets, experimental design and management

A total of 360 Shan-ma ducks at 56 days of age with similar initial body weights were randomly allocated into 3 groups: 60 birds in the low stocking density group (LSD, 5 birds/m^2^), 120 birds in the medium stocking density group (MSD, 10 birds/m^2^) and 180 birds in the high stocking density group (HSD, 15 birds/m^2^). Each group of ducks was further subdivided equally into 6 repeats, and six replicate pens with the same feeding conditions and of the same size were used. The animal experiment continued for 9 weeks. To ensure the designated SD in each group, one additional bird with similar body weights was used as replacement and added to each replicate pen to maintain the SD in the 3rd and 6th weeks of the experiment. The ducks were reared semi-openly on land and water, the number of feeding troughs and drinking water tanks in each group was controlled to ensure that the feeding level and drinking water area were the same for all ducks. The amount of feed is 60 g/d per duck in each group, and the water were supplied for free to drink. Natural ventilation was adopted in the shed, the indoor temperature was 25°C ±3°C, and the relative humidity was 75% to 85%. All the ducks were provided with the same diets, and the chemical composition of the basal diet is shown in Table 1.

### Sample collection

Six birds with body weights close to the average of each group were selected (1 bird per replicate pens) for blood sample collection in the 3rd, 6th, and 9th weeks of the experiment. The birds were subsequently euthanized for liver and kidney collection; parts of the liver and kidney samples were fixed with 4% paraformaldehyde for histopathological examination, and residuals were flash-frozen in liquid nitrogen and stored at −80°C for further use. Blood samples were collected from the wing vein and placed at 4°C for 2 h. Serum was then obtained by centrifugation at 4,000 r/min for 10 min at 4°C and stored at −20°C for further use. The fixed liver and kidney samples were processed routinely for paraffin embedding followed by sectioning and staining with hematoxylin and eosin (HE) for histopathological examination.

### Measurement of uric acid, lipopolysaccharide, and inflammatory cytokines in serum

The concentrations of UA and lipopolysaccharide (LPS) in serum were assessed by colorimetry with commercial kits (Nanjing Jiancheng Bioengineering Institute, Nanjing, China) according to the manufacturer’s protocols. The concentrations of inflammatory cytokines, including interleukin-1β (IL-1β), IL-8, and tumor necrosis factor-α (TNF-α), in serum were measured by enzyme-linked immunosorbent assay using commercial kits for ducks (Shanghai MLBIO Biotechnology Co. Ltd, Shanghai, China) according to the manufacturer’s protocols. Six bird replicates were measured in each group at every time point.

### RNA extraction and quantitative real-time polymerase chain reaction

Total RNA was extracted from the liver and kidney by TRIzol reagent (Invitrogen Inc., Carlsbad, CA, USA) and reverse transcribed to cDNA using a PrimeScript RT Reagent Kit with gDNA Eraser (Takara Bio Inc., Tokyo, Japan) according to the manufacturer’s protocols. The synthesized cDNA was stored at −20°C for gene expression tests. The mRNA expression levels of enzymes responsible for UA synthesis in the liver, including ADA, XOD, PRPPAT, and PRPS1, and the mRNA expression levels of ABCG2 and SLC2A9, which are responsible for UA excretion in the kidney, were determined by real-time quantitative polymerase chain reaction (RT-qPCR). In addition, the mRNA expression levels of tight junction-related proteins such as occludin (OCLN) and zona occludens-1 (ZO1) in the kidney were determined. Moreover, the mRNA expression levels of inflammatory cytokines (IL-8 and TNF-α) and relevant inflammatory pathway components, such as toll-like receptor 4 (TLR-4), myeloid differentiation primary response gene 88 (MyD88), and nuclear factor-κB (NF-κB), in the liver and kidney were also measured. The *β-actin* gene was used as an invariant control, and thegene expression was quantified using the 2^−ΔΔCt^ method. The primers used in this study are shown in Table 2. A 20 μL reaction volume was prepared according to the manufacturer’s protocol of the 2× RealStar Green Fast Mixture with ROX II (GenStar, Beijing, China) for real-time fluorescence quantification using the cDNA as templates. The reaction system comprised 10 μL of 2× RealStar Green Fast Mixture with ROX II, upstream and downstream primers (0.2 μL each), ddH_2_O (9.1 μL), and cDNA (0.5 μL). The PCR procedure consisted of 95°C for 2 min, all for one cycle; 95°C for 15 s, 60°C for 30 s and then 72°C for 30 s, all for 40 cycles. Three replicates were analyzed for each sample.

### Statistical analysis

Statistical analysis was performed by one-way analysis of variance using SPSS 19.0 software (SPSS Inc., Chicago, IL, USA). Data were tested for distribution normality and homogeneity of variance, and the significance of differences among treatments was tested using Tukey’s multiple comparisons test. The results are presented as the mean±standard error of the mean, with p<0.05 (*) indicating a significant difference, p<0.01 (**) and p<0.001 (***) indicating extremely significant differences, and p>0.05 indicating no significant differences.

## RESULTS

### Histopathological lesions in the liver and kidney

**Figure 1:**
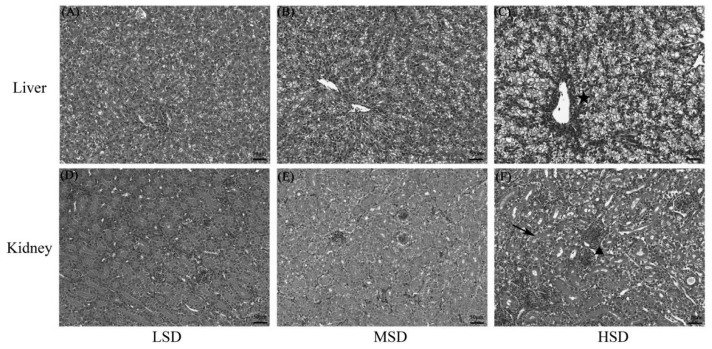
The effects of stocking densities on the histopathological lesions in the livers and kidneys of Shan-ma ducks. (A–C) Histopathological section of livers from the LSD, MSD, and HSD groups, respectively, and the star indicates the infiltration of inflammatory cells around central veins in the liver in the HSD group. (D–F) Histopathological sections of kidneys from the LSD, MSD, and HSD groups. The triangle indicates glomerular swelling and renal tubule and capsule lumen narrowing, and the black arrow shows renal tubular epithelial cells that were degenerated, necrosed and abscised from the basement membrane. LSD, low stocking density group; MSD, medium stocking density group; HSD, high stocking density group.

Samples were collected for histopathological detection in the 9th week for all three groups. The results showed that the tissue lesions for liver and kidney is absence or mild in LSD (Figure 1A, 1D) and MSD (Figure 1B, 1E) group. However, hepatocytes were acutely swollen with vacuolar degeneration, hepatocyte arrangement disorder was observed in the livers in the HSD group, and serious tissue lesions were observed around central veins with the infiltration of inflammatory cells in the HSD group (Figure 1C, ★). The renal tubule and capsule lumen were narrowed, which was accompanied by glomerular swelling (Figure 1F, ▲); the renal tubular epithelial cells degenerated, necrosed and abscised from the basement membrane (Figure 1F, black arrow); and lymphocyte infiltration was also observed in the kidneys in the HSD group. These results indicated that high SD could induce tissue lesions in the liver and kidney of ducks.

### Levels of uric acid and lipopolysaccharide in the serum

**Figure 2:**
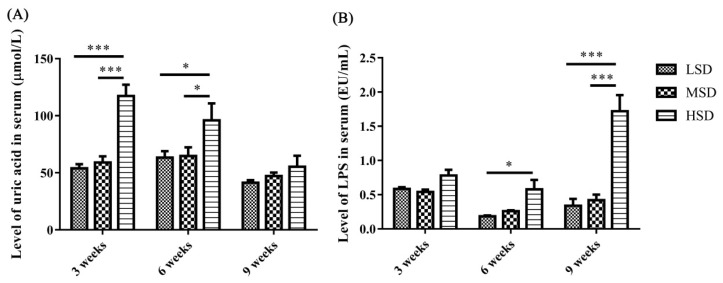
Effects of stocking densities on the levels of UA and LPS in serum of Shan-ma ducks. (A) The level of UA in serum from the LSD, MSD, and HSD groups. (B) The level of LPS in serum from the LSD, MSD, and HSD groups. Data are presented as the mean±standard error of the mean, n = 6. UA, uric acid; LPS, lipopolysaccharide; LSD, low stocking density group; MSD, medium stocking density group; HSD, high stocking density group. * p<0.05; *** p<0.001.

The level of UA in serum increased significantly (p<0.05) in the HSD group compared to the LSD and MSD groups in the 3rd and 6th weeks of the experiment (Figure 2A). In addition, the level of LPS was increased significantly (p<0.05) in the HSD group in the 6th and 9th weeks of the experiment (Figure 2B). These results indicated that high SD could affect the levels of UA and LPS in the serum of ducks, which might further affect the health or induce gout in the ducks.

### The levels of inflammatory cytokines in serum and the mRNA expression levels of cytokines in liver and kidney

**Figure 3:**
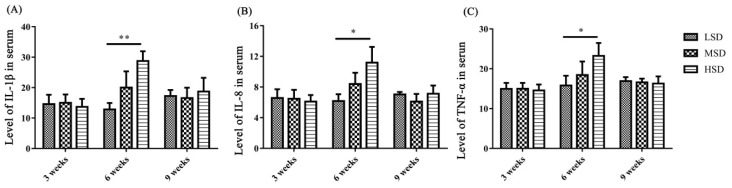
The levels of inflammatory cytokines (IL-1β, IL-8, and TNF-α) in the serum of Shan-ma ducks under different stocking densities. (A) The level of IL-1β in serum from the LSD, MSD, and HSD groups. (B) The level of IL-8 in serum from the LSD, MSD, and HSD groups. (C) The level of TNF-α in serum from the LSD, MSD, and HSD groups. Data are presented as the mean±standard error of the mean, n = 6. IL-1β, interleukin-1β; TNF-α, tumor necrosis factor-α; LSD, low stocking density group; MSD, medium stocking density group; HSD, high stocking density group. * p<0.05; ** p<0.01.

To determine the reasons for the histopathological lesions in the liver and kidney, the levels of inflammatory cytokines in serum and the mRNA expression levels of inflammatory cytokines and related pathway components in the liver and kidney were measured. The levels of IL-1β, IL-8, and TNF-α in serum were significantly increased in the HSD group (p< 0.05) compared to the LSD group in the 6th week of the experiment (Figure 3). However, there were no significant changes in serum IL-1β, IL-8, and TNF-α levels in the 3rd and 9th weeks of the experiment.

**Figure 4:**
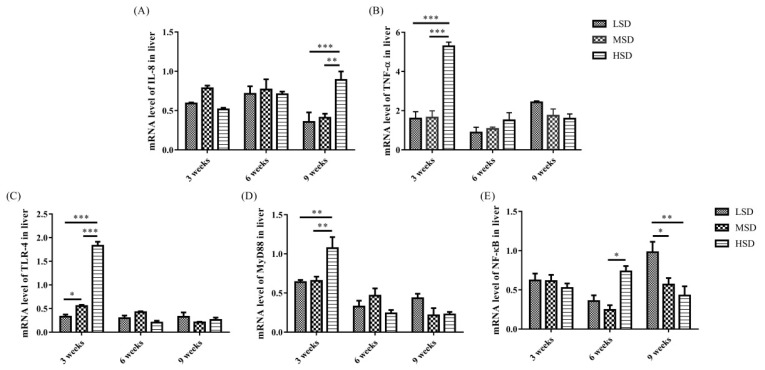
The mRNA expression levels of inflammatory cytokines (IL-8 and TNF-α) and related pathway components (TLR-4, MyD88, and NF-κB) in the livers of Shan-ma ducks under different stocking densities. Data are presented as the mean±standard error of the mean, n = 6. IL-8, interleukin-8; TNF-α, tumor necrosis factor-α; TLR-4, toll-like receptor 4; MyD88, myeloid differentiation primary response gene 88; NF-κB, nuclear factor-κB. * p<0.05; ** p<0.01; *** p<0.001.

**Figure 5:**
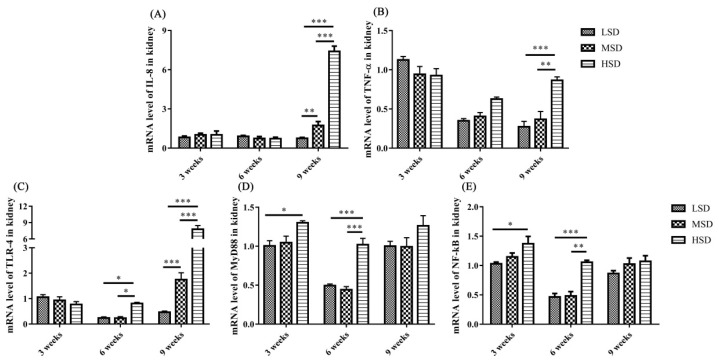
The mRNA expression levels of inflammatory cytokines and related pathway components in the kidneys of Shan-ma ducks under different stocking densities. Data are presented as the mean±standard error of the mean, n = 6. * p<0.05; ** p<0.01; *** p<0.001.

The mRNA expression level of IL-8 was increased significantly (p<0.01) in the livers in the HSD group in the 9th week and the mRNA expression level of TNF-α increased significantly (p<0.01) in the livers in the HSD group in the 3rd weeks of the experiment (Figure 4A, 4B), respectively. In addition, the mRNA expression levels of TLR-4, MyD88, and NF-κB were increased significantly (p<0.01) in the livers in the HSD group in the 3rd or 6th weeks of the experiment (Figure 4C–4E). However, the expression of NF-κB was decreased significantly (p<0.05) in the 9th week of the experiment in the HSD group (Figure 4E). For the kidney, the mRNA expression levels of IL-8 and TNF-α were increased significantly (p<0.01) in the HSD group in the 9th week of the experiment (Figure 5A, 5B). In addition, the mRNA expression levels of TLR-4, MyD88, and NF-κB were also increased significantly (p<0.05) in the kidneys in the HSD group (Figure 5C–5E). These results indicated that the ducks suffered inflammatory responses in both the liver and kidney under high SD, which further induced serious histopathological lesions in these tissues.

### The mRNA expression levels of enzymes responsible for the synthesis of uric acid in the liver

**Figure 6:**
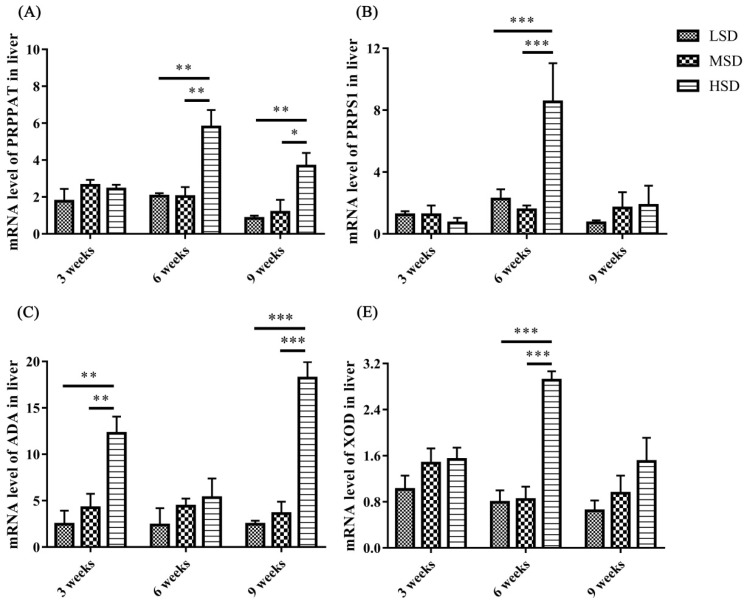
The mRNA expression levels of enzymes (ADA, XOD, PRPPAT, and PRPS1) related to the synthesis of UA in the livers of Shan-ma ducks under different stocking densities. Data are presented as the mean±standard error of the mean, n = 6. ADA, adenosine deaminase; XOD, xanthine oxidase; PRPPAT, phosphoribosyl pyrophosphate amidotransferase; PRPS1, phosphoribosyl pyrophosphate synthetase1; UA, uric acid. * p<0.05; ** p<0.01; *** p<0.001.

To determine the possible reasons for the increase in UA in serum, the mRNA expression levels of enzymes responsible for the synthesis of UA in the liver were measured, and the results showed that the mRNA expression level of ADA was increased significantly (p<0.001) in the HSD group in tne 3rd and 9th weeks of the experiment compared to the LSD and MSD groups (Figure 6C). The mRNA expression levels of XOD, PRPPAT, and PRPS1 were increased significantly (p<0.01) in the 6th or 9th weeks of the experiment (Figure 6). These results indicated that high SD could affect the gene expression levels of enzymes responsible for the synthesis of UA and further lead to an increase in UA in serum in ducks.

### The mRNA expression levels of proteins responsible for the excretion of uric acid in the kidney

**Figure 7:**
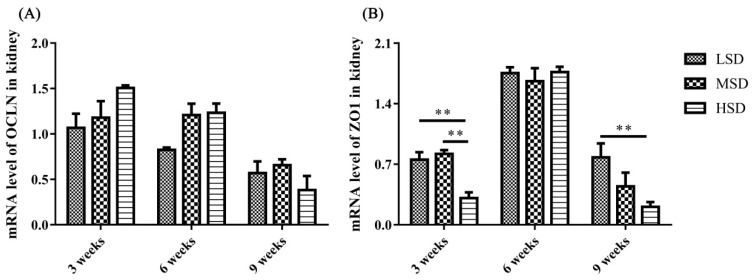
The mRNA expression levels of tight junction proteins in the kidneys of Shan-ma ducks. (A) The mRNA expression level of OCLN in the kidneys from the LSD, MSD, and HSD groups. (B) The mRNA expression level of ZO1 in the kidneys from the LSD, MSD, and HSD groups. Data are presented as the mean±standard error of the mean, n = 6. OCLN, occludin; LSD, low stocking density group; MSD, medium stocking density group; HSD, high stocking density group; ZO1, zona occludens-1. ** p<0.01.

**Figure 8:**
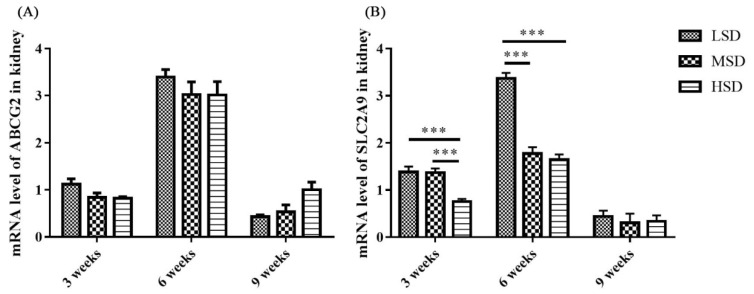
The mRNA expression levels of ABCG2 and SLC2A9 are responsible for the excretion of UA by the kidneys of Shan-ma ducks. (A) The mRNA expression level of ABCG2 in the kidneys from the LSD, MSD, and HSD groups. (B) The mRNA expression level of SLC2A9 in the kidneys from the LSD, MSD, and HSD groups. Data are presented as the mean±standard error of the mean, n = 6. ABCG2, ATP-binding cassette subfamily G member 2; SLC2A9, solute carrier family 2 member 9; LSD, low stocking density group; MSD, medium stocking density group; HSD, high stocking density group. *** p<0.001.

The mRNA expression level of tight junction-related preteins were determined, and the results showed that the mRNA expression level of ZO1 was decreased significantly in the HSD group in the 3rd and 9th weeks of the experiment (Figure 7). The kidney is responsible for the excretion of UA, which is controlled by a suite of apically and basolaterally expressed secretory and reabsorptive molecules, such as ABCG2 and SCL2A9. As shown in Figure 8, the mRNA expression level of ABCG2 in the kidney was not significantly different among the three groups (p>0.05). However, the mRNA expression level of renal SLC2A9 was decreased significantly (p<0.001) in the HSD group in the 3rd and 6th weeks of the experiment (Figure 8B), which indicated that the inhibition of UA excretion might further induce the increase in UA in serum.

## DISCUSSION

Studies on the effects of SD on poultry health and production, such as decreasing growth performance, meat quality, intestinal barrier function, and immunity, have been reported in recent years [1,7]. However, the effects of SD on the metabolism of UA and the related functions of the liver and kidney have not been reported, and these issues are the focus of the present study. Higher SD means higher fecal excretion of pathogenic bacteria and nutritious substances of nitrogen and phosphorus, which could exacerbate bacterial proliferation and LPS contamination in water [24]. Previous studies have found that the number of *Escherichia coli*,* Salmonella* and total bacteria in water under high SD increased significantly, with increasing LPS concentration in water and in the blood of waterfowl [24]. In the present study, the concentration of LPS in serum increased significantly in the HSD group (Figure 2), which might be caused by the large amount of bacterial proliferation in the HSD group. In addition, inflammatory cytokines, including IL-1β, IL-8, and TNF-α, in serum were also increased significantly in the HSD group at the 6th week (Figure 3), However, there is no significant difference for these inflammatory cytokines in serum among three groups at the 9th week, which might because the stress-induced negative immunomodulation under long-term high SD conditions as reported previously [25].

The increase in inflammatory cytokines may be caused by the accumulation of LPS in the ducks in the HSD group, and it induced the activation of TLR/MyD88/NF-κB signaling pathways and increased the production of proinflammatory cytokines. Indeed, the mRNA expression levels of TLR-4, MyD88, and NF-κB, as well as IL-8 and TNF-α, were increased in both the livers and kidneys in the HSD group (Figure 4–5), indicating the activation of the TLR-4/MyD88/NF-κB signaling pathways. However, the expression of NF-κB was decreased in the 9th week of the experiment in the HSD group, which may be caused by immunosuppression under long-term high SD conditions [25]. In addition, tissue lesions and infiltration of inflammatory cells were observed in both the liver and kidney. These results indicated that high SD could cause inflammatory responses and further induce tissue lesions in the livers and kidneys of ducks.

Avian gout is a common metabolic disease characterized by HUA and urate deposits in the viscera or joints [13], and HUA is caused by the overproduction of UA by hepatic metabolism or renal underexcretion [26,27]. Lesions in the liver and kidney, as well as increased expression levels of enzymes related to the synthesis of UA, contribute to HUA and gout formation [14,17]. XOD, which is highly expressed in the liver, is responsible for the synthesis of UA and oxidizes xanthine and hypoxanthine into UA, and ADA, PRPPAT, and PRPS1 can promote the production of xanthine and hypoxanthine [14,16]. In the present study, the mRNA expression levels of ADA, XOD, PRPPAT, and PRPS1 in the liver were increased significantly in the HSD group compared to those in the other two groups (Figure 6). These results demonstrated that high SD could increase the synthesis of UA by promoting the mRNA expression of enzymes responsible for the synthesis of UA in the liver. In addition, the level of UA in serum also increased significantly at the 3rd and 6th weeks in the HSD group, which might further induce HUA and gout formation. However, there is no significant difference in the UA level was observed at the 9th week among three groups, this might because the recovery of the excretion fuction for kidney, which can be reflected from the mRNA expression level of SLC2A9 at the 9th week.

Studies on the pathogenesis of avian gout indicated that the renal underexcretion of UA is another important factor related to gout [28]. Many factors that induce kidney lesions could lead to renal underexcretion of UA, such as virus infection, LPS and drug or toxin toxicosis [13,14,29]. ABCG2 and SLC2A9 have been reported to play important roles in the regulation of UA [16,19], and SLC2A9 is the key pathway of urate reabsorption and excretion from the proximal tubule in the kidney [15,20]. The mRNA expression level of ABCG2 in the kidney showed no significant difference in the present study, which was similar to the findings from previous studies on gout in goslings [13]. However, the renal mRNA expression level of SLC2A9 decreased significantly in the HSD group, which probably led to the underexcretion of UA in the kidney. In addition, tight junction proteins are intercellular adhesion complexes that are essential for the barrier function of epithelia and are important for permeability; these proteins include a series of transmembrane proteins, such as claudins (CLDNs), OCLN, ZO1 and so on [30]. The mRNA expression level of ZO1 was decreased significantly in the HSD group in the 3rd and 9th weeks of the experiment. Combined with the histopathological changes observed in the kidney sections, it was thoguht that the permeability and integrity of renal epithelial cells were disrupted, which might be caused by the increase in LPS in serum, and further induced the renal underexcretion of UA in the HSD group.

## CONCLUSION

In conclusion, the present study revealed that high SD could induced a higher expression level of inflammatory cytokine and cause tissue inflammatory lesions in the liver and kidney, and subsequently affect the metabolism of UA in liver and the excretion of UA in kidney. These results presented in this study is helpful for guiding decisions related to the breeding and production of ducks under an intensive culture model.

## Figures and Tables

**Table 1:** Composition and nutrient level of basal diet (air-dry basis)

Items	Content %
Ingredients	
Corn	60.00
Wheat bran	11.45
Soybean meal	24.00
Limestone	1.02
Soybean oil	0.50
Salt	0.30
Lysine	0.01
L-Methionine	0.12
Calcium bicarbonate	1.60
Premix^1)^	1.00
Total	100.00
Nutrient levels^2)^	
Metabolizable energy^3)^ (MJ/kg)	11.00
Crude protein	18.00
Calcium	1.00
Total phosphorus	0.57
Available phosphorus	0.33
Methionine	0.40
Lysine	0.87

1) Premix provided per kilogram of diet: Vitamin A 12,000 IU, Vitamin B_1_ 1 mg, Vitamin B_2_ 8 mg, Vitamin B_6_ 6 mg, Vitamin B_12_ 0.1 mg, Vitamin D_3_ 2,500 IU, Vitamin E 25 mg, Vitamin K_3_ 2 mg, biotin 0.3 mg, niacin 50 mg, pantothenic acid 15 mg, folic acid 1 mg, Zn 80 mg, Mn 80 mg, Cu 10 mg, Fe 50 mg, I 0.1 mg, Se 0.5 mg.

2) Calculated Values.

3) The values are calculated according to the AME of ducks.

**Table 2:** Primer sequences for real-time quantitative polymerase chain reaction

Gene name	Nucleotide sequence (5′ – 3′)	Product length (bp)	GenBank accession number
*β-actin*	F: GGTATCGGCAGCAGTCTTA R: TTCACAGAGGCGAGTAACTT	160	XM_013174886.1
*ADA*	F: TATTCGATAAACACCGATGACCC R: TCACTCTCTTGAAATCCTCGT	109	XM_013188525.1
*XOD*	F: GGGGAAGATGGTGAGATGGA R: ACGATGCGATTTGATGGGAC	101	NM_205127.2
*PRPPAT*	F: CAAACGCTGGATGTGGTA R: AGACTCTGGAACGGTGCT	192	XM_038178494.1
*PRPS1*	F: GTAGTCACAAACACAATACCCC R: ACAGATTCCCCATTATGAGTCCT	119	XM_027465168.2
*ABCG2*	F: GACATCATCAACGGGGACTC R: ACTTCTCAGCCAAGGTTGTGT	112	XM_005012770.5
*SLC2A9*	F: CAAAGTTTTCTTGGGCACCT R: CACACCCCACCATTACCAC	131	XM_027457388.2
*OCLN*	F: CGCCACGTTCTTCACCCACTC R: CTCATCTGCTTCTTCGCCCACA	129	XM_013199669.1
*ZO1*	F: CTTCAGGTGTTTCTCTTCCTCCTC R: CTGTGGTTTCATGGCTGGATC	131	XM_040680632.1

*ADA*, adenosine deaminase; *XOD*, xanthine oxidase; *PRPPAT*, phosphoribosyl pyrophosphate amidotransferase; *PRPS1*, phosphoribosyl pyrophosphate synthetase1; *ABCG2*, ATP-binding cassette subfamily G member 2; *SLC2A9*, solute carrier family 2 member 9; *OCLN*, occludin; *ZO1*, zona occludens-1.
